# Quantifying Rock Weakening Due to Decreasing Calcite Mineral Content by Numerical Simulations

**DOI:** 10.3390/ma11040542

**Published:** 2018-04-01

**Authors:** Maria Wetzel, Thomas Kempka, Michael Kühn

**Affiliations:** 1 Fluid Systems Modelling, GFZ German Research Centre for Geosciences, Telegrafenberg, 14473 Potsdam, Germany; thomas.kempka@gfz-potsdam.de; 2 Institute of Earth and Environmental Science, University of Potsdam, Karl-Liebknecht-Str. 24/25, 14476 Potsdam, Germany; michael.kuehn@uni-potsdam.de

**Keywords:** digital rock physics, micro-CT, elastic properties, numerical simulation, chemical-mechanical interaction, Code_Aster, composite properties

## Abstract

The quantification of changes in geomechanical properties due to chemical reactions is of paramount importance for geological subsurface utilisation, since mineral dissolution generally reduces rock stiffness. In the present study, the effective elastic moduli of two digital rock samples, the Fontainebleau and Bentheim sandstones, are numerically determined based on micro-CT images. Reduction in rock stiffness due to the dissolution of 10% calcite cement by volume out of the pore network is quantified for three synthetic spatial calcite distributions (coating, partial filling and random) using representative sub-cubes derived from the digital rock samples. Due to the reduced calcite content, bulk and shear moduli decrease by 34% and 38% in maximum, respectively. Total porosity is clearly the dominant parameter, while spatial calcite distribution has a minor impact, except for a randomly chosen cement distribution within the pore network. Moreover, applying an initial stiffness reduced by 47% for the calcite cement results only in a slightly weaker mechanical behaviour. Using the quantitative approach introduced here substantially improves the accuracy of predictions in elastic rock properties compared to general analytical methods, and further enables quantification of uncertainties related to spatial variations in porosity and mineral distribution.

## 1. Introduction

**Figure 1:**
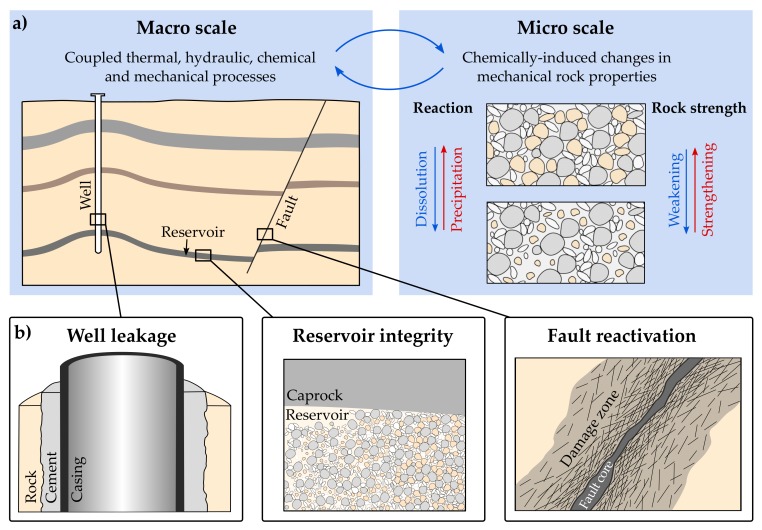
(**a**) Chemical processes at micro scale, as the precipitation and the dissolution of minerals, can significantly weaken or strengthen a rock, which affects coupled processes at the macro scale. (**b**) This is of particular relevance for risk assessment within the context of geological subsurface utilisation, e.g., to assess potential well leakage, reservoir integrity and fault reactivation.

Mineral dissolution and precipitation are micro-scale processes, which may significantly change the mineralogical composition and microstructure of rocks, and consequently affect the effective mechanical rock behaviour at the macro scale. Predicting these changes has a wide practical importance in applied sciences and materials engineering, especially for hydraulically conductive materials where reactive transport takes place (Figure 1): hydrothermal alterations may weaken geological fractures [1] and fault zones up to their reactivation [2], chemical degradation of cements may influence the integrity of wells [3,4] and mineral dissolution can lead to significant weakening of geological reservoirs [5,6]. Hence, the quantification of this direct chemical–mechanical interaction is of substantial relevance within the context of risk assessment for most applications related to geological subsurface utilisation such as geothermal energy systems [7,8,9], enhanced oil recovery [10,11], radioactive waste disposal [12,13], underground coal gasification [14,15,16] and CO_2_ or geological energy storage [17,18,19].

The main factors contributing to macro-scale elastic rock properties are microstructure and mineral volume fractions as well as the constituent moduli. In addition to the experimental determination of elastic parameters due to changes in rock composition [5,20], a variety of analytical and numerical methods for their calculation exists (Appendix A). Relatively simple approximations are bounding methods, which predict upper and lower limits of the moduli for any given composition, as the estimates introduced by Voigt [21] and Reuss [22], respectively. Both methods average parameters based on the volume fractions of the rock constituents, while the narrower Hashin-Shtrikman [23] bounds allow additional consideration of a spherical shape of these components. The soft-sand and its counterpart the stiff-sand model [24] are based on sphere pack assumptions [25,26] and further take into account grain cementation. Both models are used for granular high-porosity rocks [27], being limited to only two components: fluid and rock. Traditional mean-field homogenisation schemes have the advantage of considering ellipsoidal inclusions of multiple components and are used to address several rock types [28,29,30,31]. These schemes are based on Eshelby’s solution [32] of one ellipsoidal inhomogeneity embedded in an infinite medium and involve the dilute scheme [33], the self-consistent approximation [34] as well as the Mori-Tanaka approach [35]. The simple dilute scheme neglects any mechanical interaction between neighbouring inclusions, and is therefore only valid for low-volume fractions. The Mori-Tanaka approach considers the interaction between the inclusions, but their effects on matrix properties are neglected. Therefore, it is only valid for inclusion volume fractions of up to 20–30% [36,37], whereas the self-consistent approximation is valid for higher inclusion volume fractions [38], since it considers no clear matrix phase and the effective medium is assumed to be a component itself. However, none of the analytical models can determine elastic rock parameters for multi-component systems over a broad range of volume fractions and complex rock microstructures [39,40,41].

**Figure 2:**
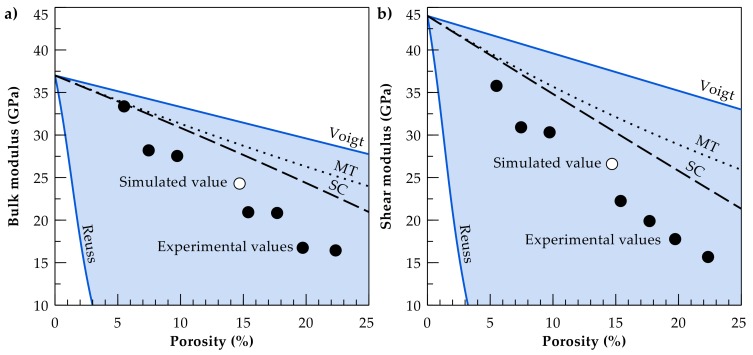
State-of-the-art analytical solutions as the Mori-Tanaka approach (MT, dotted line) and the self-consistent scheme (SC, dashed line) overestimate the experimentally determined (**a**) bulk and (**b**) shear moduli (black-filled circles) of the Fontainebleau sandstone [64], while numerical simulations (empty circle) based on a micro-CT scan [59] result in substantially better estimations.

Despite their higher computational demand, numerical models are more flexible regarding the number of constituents and complex geometries. These are further used to quantify the influence of pore network and inclusion geometries, using artificial microstructures [42,43,44] or simulating sedimentation processes [45]. Further, petrophysical properties of rocks can be calculated based on 3D images derived from micro-computer tomography (micro-CT) scans. The resulting model geometries consider a more realistic microstructure regarding the shape of single grains, cements and the overall pore network [46,47]. Digital rock physics in view of a virtual laboratory have the advantage of offering a non-destructive method with immediate access to rock parameters. Moreover, the same rock sample can be examined under varying testing conditions or mineral composition, which can be virtually varied [48]. Present numerical models employ calculation techniques to determine static [49,50,51] and dynamic moduli [52] mainly for reservoir rocks such as sandstones [53,54] and carbonates [55,56], but also for shales [57]. However, there is still a discrepancy between the achieved numerical estimates of mechanical properties derived from micro-CT images and laboratory data, where, regardless of the numerical approach, the simulated moduli are systematically higher [58,59]. Uncertainties resulting from micro-CT image processing and the applied segmentation method may contribute to these deviations. Another reason for this numerical overestimation is the inability to fully characterise micro heterogeneities such as grain contacts, small-scale pores and crack networks [60,61]. Furthermore, the effective properties of the mineral phases may be significantly lower than those listed in Mavko et al. [27], as illustrated by Mahabadi et al. [62] who determined the mean Young’s modulus of quartz by micro-indentation testing and demonstrated that its actual value is 15 GPa below that in the standard table [27]. Nevertheless, improving the computational workflow is ongoing research in digital rock physics [60,63], with numerical calculations providing already better estimates of bulk (K) and shear moduli (G) than analytical solutions for certain microstructures (Figure 2).

The present study employs a numerical approach to quantify changes in elastic rock properties, resulting from assumed mineralogical changes within the microstructure. For that purpose, 3D models of two sandstones are used, whereby calcite cement precipitated in the pore network is successively reduced. Since detailed micro-CT data on the development of the spatial calcite distribution in a reference volume are not yet publicly available, the general effect of its spatial distribution is examined by three different geometrical arrangements, including a random distribution, partial filling and coating around the grains. In this context, we also considered the impact of initial elastic moduli of the calcite cement. The simulated mechanical parameters are discussed in the context of widely used state-of-the-art analytical solutions. Improving the quantification of changes in elastic properties as well as the determination of uncertainties related to spatial variations in porosity and mineral distribution are the objectives of our study, supported by an innovative computational method. The investigated scenario is especially of interest within the context of geological subsurface utilisation at operational time scales (e.g., 30–50 years), where changes in elastic properties are of particular relevance to assess reservoir, caprock and fault integrity. Moreover, our findings can be directly applied in material sciences.

## 2. Materials and Methods

### 2.1. Characterisation of the Digital Sandstone Samples

**Figure 3:**
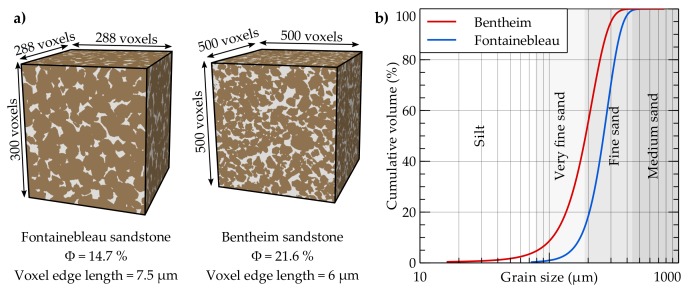
(**a**) Size and resolution of the binary micro-CT scans for the digital sandstone samples [59,70]. The voxel edge length of the original Bentheim microstructure is reduced to 6 μm to achieve a grain resolution equivalent to the Fontainebleau microstructure. (**b**) Both sandstones have similar grain size distributions, while the Bentheim sandstone has a slightly wider distribution in particle size.

In the present study, digital rock samples of the Fontainebleau and Bentheim sandstones are used, which have been comprehensively examined as reference rocks for reservoir engineering applications [65,66]. The available binary datasets are segmented and comprise only grains and intergranular porosity. Both rocks represent fine-grained sandstones (Figure 3b) and are homogeneous and isotropic at the investigated scale [67]. The quasi-pure Fontainebleau sandstone consists of well-sorted monodisperse quartz grains [68], whereby its elastic properties have been studied over a wide range of porosities [64]. The microstructure of the Fontainebleau sample employed here derives from the digital rock physics benchmark of Andrä et al. [59] and has a resolution of 288 × 288 × 300 voxels with an edge length of 7.5 μm (Figure 3a). The total porosity of this digital sample is 14.7%, with a mean grain diameter of 260 μm, calculated by means of the image processing software package ImageJ [69] using a watershed segmentation and particle analysis. The Bentheim sandstone consists besides its main constituent quartz of the minor components kaolinite (3%) and orthoclase (2%) [65]. Nevertheless, the provided microstructure by Alhashmi et al. [70] only comprises a binary dataset. With 1000^3^ voxels and an edge length of 3 μm, it has a considerably higher resolution than the digital Fontainebleau sample. Further, the Bentheim sandstone has a higher porosity (21.6%), a lower mean grain size (190 μm) and a slightly wider particle size distribution (Figure 3b). Since effective elastic moduli are considerably influenced by rock microstructure [52] and differences due to micro-CT resolution should be avoided, the comparability of the microstructures is achieved by an equivalent voxel size, which is scaled to the average grain size. For the Fontainebleau sandstone, 35 voxels characterise an average grain, while the overall resolution of the Bentheim sample has to be reduced to 500^3^ voxels with an edge length of 6 μm to ensure a comparable representation of 32 voxels per grain.

### 2.2. Numerical Determination of Effective Rock Properties

**Figure 4:**
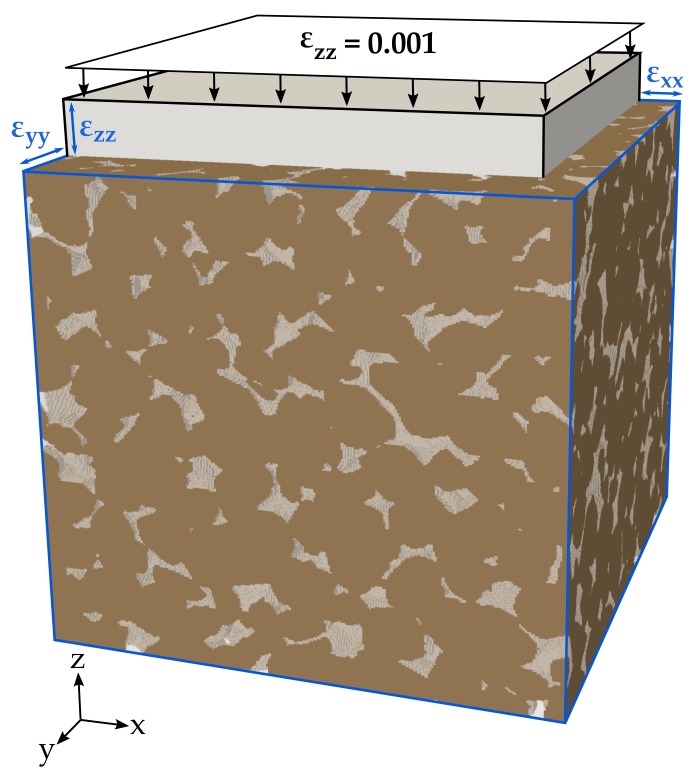
Effective elastic properties are calculated using the stress-strain behaviour of the normalized digital rock sample. For that purpose, a small strain is applied at one boundary face (z = 1), while roller boundaries fix the displacements perpendicular to its opposite face (z = 0) and two adjacent boundaries (x = 0, y = 0).

The effective elastic rock properties are computed with the open source finite element software package Code_Aster [71] by performing digital uniaxial compression tests on the 3D microstructure of the previously introduced sandstone samples. Here, static rock moduli are determined by solving the equations of linear elasticity. Both, static [38,44] and dynamic approaches [55,60] are applicable to materials with heterogeneities and produce similar results [52]. For digital uniaxial compression testing, orthogonally-mixed boundary conditions are used [72,73]. A small uniform normal strain of 0.1% of the sample’s edge length is assigned at one boundary face, while displacements perpendicular to its opposite face and two adjacent boundaries are prohibited. Thereby, the sample is compressed and strain is forced to develop in the two unconstrained directions (Figure 4). Young’s modulus and Poisson’s ratio are calculated considering the mean stresses and strains along the boundary faces of the cubic sample. Elastic parameters for each orientation are obtained by loading the sample in the respective spatial direction, whereby potential anisotropy can also be determined. Unless otherwise noted, the effective elastic moduli are the mean values, resulting from the three different orthogonal loading directions. The initial parameters of the minerals are standard values, listed in Table 1. Pore space is modelled under dry conditions to ensure comparability with the data provided by Andrä et al. [59].

### 2.3. Choice of Representative Sub-Cubes

**Figure 5:**
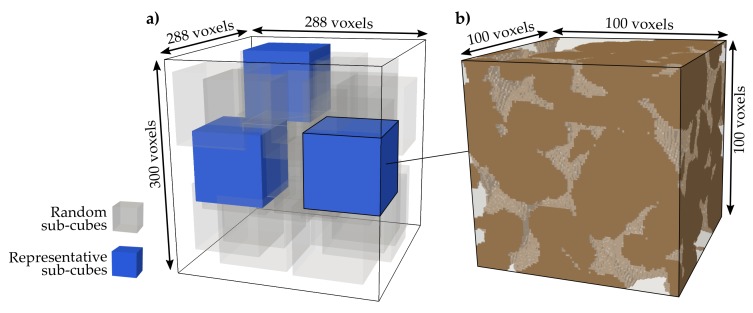
(**a**) The entire microstructure of the Fontainebleau sandstone consists of 24.8 million voxels and is randomly decomposed into 30 sub-cubes (grey) (**b**) comprising 1 million voxels each. Three sub-cubes are identified (blue) being representative for the main microstructure and are further used in our simulations to quantify changes in elastic rock properties due to variations in the calcite volume fraction.

A sub-sampling method is used to decompose the microstructure in a specific number of sub-cubes, which all together represent the main structure (Figure 5). This workflow improves the processing efficiency and results in substantially lower computational times, which is especially relevant for multi-million voxel micro-CT scans. Microstructures are often divided into regular adjacent cubes, which are separated [53] or overlapping each other [55], but also non-neighbouring sub-volumes or a random selection can be employed for that purpose [75,76]. In our approach, the Fontainebleau sandstone is randomly decomposed into 30 sub-cubes of 100^3^ voxels each, with an allowed overlap of 34% in maximum. Since we calculate the effective elastic properties on the full micro-CT scan resolution, the employed methodology is validated against the benchmark of Ändrä et al. [59], who used the method of Garboczi [77] to determine the elastic parameters. In this context, they determined a bulk modulus of 24.3 and shear modulus of 26.6 GPa, while our mean values are 24.8 and 26.1 GPa, respectively (Table 2). The considerably larger microstructure of the Bentheim sandstone is decomposed randomly into 60 non-overlapping sub-cubes under consideration of the same grain resolution as applied for the Fontainebleau sandstone. Thus, 48% of the rock volume is sub-sampled. Calculating the mechanical properties only for a part of the whole sample will give reasonable results, since Faisal et al. [78] proposed that sampling of a volume fraction of 40–55% by non-overlapping cubes is sufficient to determine effective moduli. The resulting elastic properties and porosities vary for each of the sub-cubes and are discussed in Section 3.1. By decomposing the whole structure in smaller sub-volumes, three representative sub-cubes are identified for each rock. These sub-cubes are chosen by means of two criteria: (1) isotropy of elastic rock properties and (2) range of porosity variation. Both criteria support the representation of the range of uncertainty based on the rock microstructure. The resulting sub-cubes are used for our subsequent simulations to assess the change in elastic rock properties due to the removal of calcite cement.

### 2.4. Dissolution of Calcite Cementation

**Figure 6:**
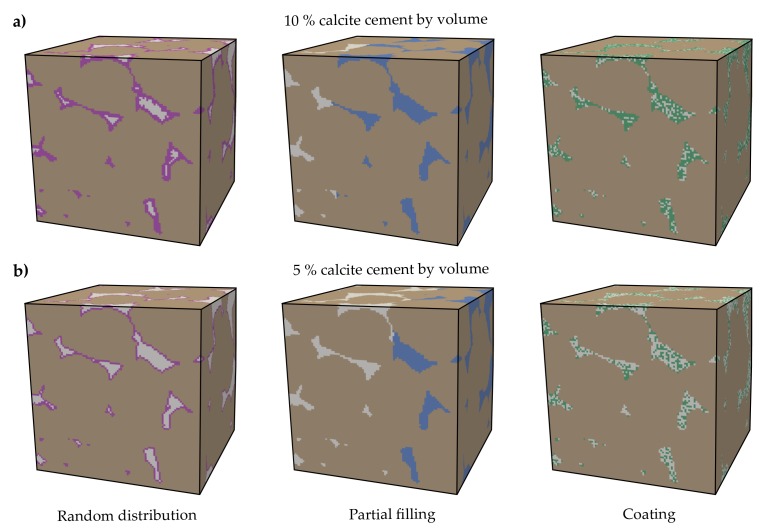
Representative sub-cubes of the Fontainebleau sandstone comprise quartz grains (brown), pore space (light grey) and calcite cement with three general spatial arrangements: random distribution (green), partial filling (blue) and coating (magenta). The pore network is exemplarily filled with a calcite cement volume fraction of (**a**) 10% and (**b**) 5%.

Former simulations, which investigated variations in elastic properties due to rock alteration, either assumed changes in its microstructure [75,79] or used micro-CT scans in advance of and subsequent to a dissolution experiment [56]. In all cases, only binary systems have been investigated, focussing on the porosity development due to solid phase degradation. Since quartz is hardly soluble at operational timescales in geological subsurface utilisation, we consider a mineral system consisting of three components: quartz grains, calcite cement and pore space. In this context, precipitated calcite cement within the pore network of the initial digital sandstone samples is assumed, as calcite has a high solubility, fast reaction kinetics and is almost ubiquitous in sedimentary rocks. Consequently, dissolution of calcite cement can lead to substantial rock alteration and weakening. Since reactive transport simulations at pore network scale are out of the scope of the present study, three different approaches to address the dissolution of calcite cement are employed and compared here. The pore space of the two initial sandstone microstructures is filled with calcite cement up to a volume fraction of 10%, whereby the sandstone is reinforced compared to the original microstructure. The permeability of the Fontainebleau sandstone amounts to about 10 mD for an effective porosity of 5% by means of the Kozeny–Carman relation [66]. Even though this represents a relatively low reservoir permeability, the rock is not impervious to fluid flow. Further, the impact of spatial cement distribution is investigated by three general calcite arrangements, which are straightforward to implement: (1) random distribution, (2) partial filling and (3) coating around the grains (Figure 6a). These three basic geometries are used to quantify the general impact of the spatial calcite distribution and to assess if a more complex precipitation pattern, i.e., generated by reactive transport simulations at pore scale, is required for the purpose of the present study. The relative changes in effective elastic properties are quantified by numerical simulations, considering a successive removal of the hypothetical calcite cement (Figure 6b). For some binary microstructures, weaker moduli of the mineral phase quartz are assigned to the grain contact cements, so that the simulated values become consistent with the measured ones [80]. Since the calcite mineral is comparably stiff, the intra-porous calcite cement bulk and shear moduli likely notably differ from those of the mineral phase. Possible reasons for this may be different mineral structures (crystalline, amorphous) and the size of the precipitates, and therefore undetected microporosity. As data on elastic behaviour of calcite cement is not yet available apart from the stiffness moduli for the calcite mineral, a significantly softer value for amorphous calcite [74] is used in our simulations (Table 1). Both values are regarded as upper and lower bounds to enclose a possible range of effective elastic property variation and allow to examine the effect of initial cement moduli.

## 3. Results

### 3.1. Evaluation of the Representative Sub-Cubes

**Figure 7:**
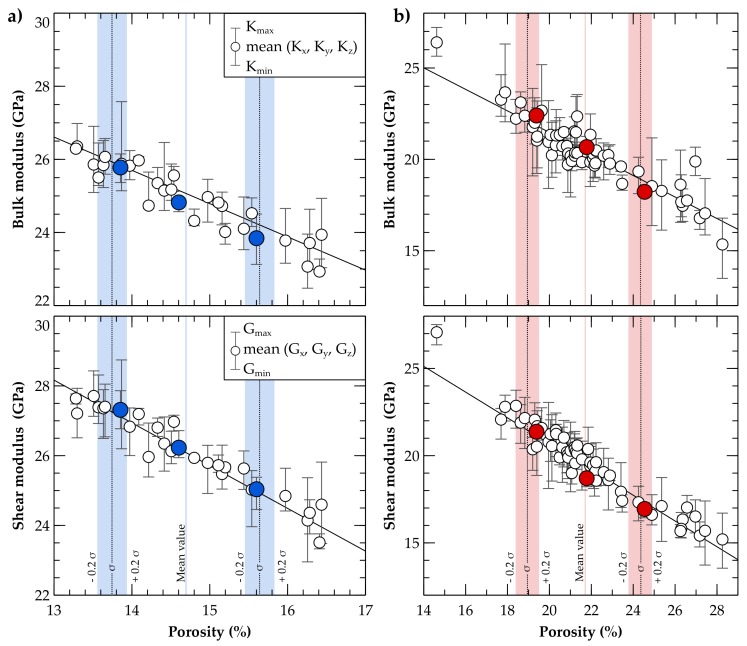
Bulk and shear moduli of all sub-cubes (empty circles) decrease linearly with porosity. Three representative sub-cubes (blue- and red-filled circles), which are preferably isotropic, are selected from (**a**) 30 Fontainebleau and (**b**) 60 Bentheim sandstone sub-samples. While the sub-volumes of the Fontainebleau sample have a relatively narrow porosity spectrum (blue lines), the range for the Bentheim sandstone porosity (red lines) is substantially wider.

The sub-samples representing the entire digital rock vary in porosity, whereby bulk and shear moduli decrease quasi-linearly with increasing porosity. Porosities of the Fontainebleau sub-cubes range between 13.3% and 16.4% (Figure 7a), while the Bentheim sample has a considerably broader porosity spectrum with 14.6% to 28.3% (Figure 7b). The calculated mean elastic properties considering all sub-cubes of the Fontainebleau sandstone show values of 24.8 and 26.1 GPa for bulk and shear moduli, respectively (Table 2), whereas the higher porosities of the Bentheim sample result in lower elastic parameters of 20.4 GPa (K) and 19.6 GPa (G). The mean directional difference of all sub-samples is determined by the mean difference between the minimum and maximum moduli for the three spatial loading directions. Higher values for the Bentheim sandstone are related to its higher total porosity and broader range in particle size as discussed in Section 2.1. The linear relationship between porosity and effective elastic properties for the representative sub-cubes is indicated by a coefficient of determination (r^2^) of 0.84 to 0.93 for both digital rocks (Table 2). Considering the moduli resulting from the different loading directions instead, characterising the anisotropy of the sub-cubes leads to a lower coefficient of determination with 0.65 to 0.79. As previously stated, the representative sub-cubes should ideally be isotropic and comprise the entire porosity variation bandwidth of the sample to support the representation of the uncertainty range by means of the rock microstructure. Hence, one sub-cube with a porosity equivalent to the entire digital rock sample is selected, while the porosities of the two other sub-volumes are within a distance of one standard deviation (σ) with a tolerance of ± 0.2 σ (Figure 7a,b). Isotropic elastic behaviour of the three sub-cubes is achieved by selecting those with the least difference in directional moduli. Based on these selection criteria, the representative sub-cubes for each rock are highlighted in Figure 7, and represents the porosity bandwidth for the entire sample. For the Fontainebleau sandstone, the elastic moduli of the representative sub-cubes differ between 25.8 and 23.8 GPa (K) as well as 27.3 and 25 GPa (G) (Table 3). The mean value of the three selected sub-cubes deviates between 2.1% (K) and 1.6% (G) compared to the calculated values of Andrä et al. [59] for the entire Fontainebleau microstructure, what demonstrates the validity of our results and the approach using representative sub-cubes to calculate effective properties. The range is broader for the representative sub-volumes of the Bentheim sandstone with 22.4 to 18.2 GPa and 21.4 to 16.9 GPa for bulk and shear moduli, respectively.

### 3.2. Effect of Spatial Calcite Distribution on Elastic Rock Properties

**Figure 8:**
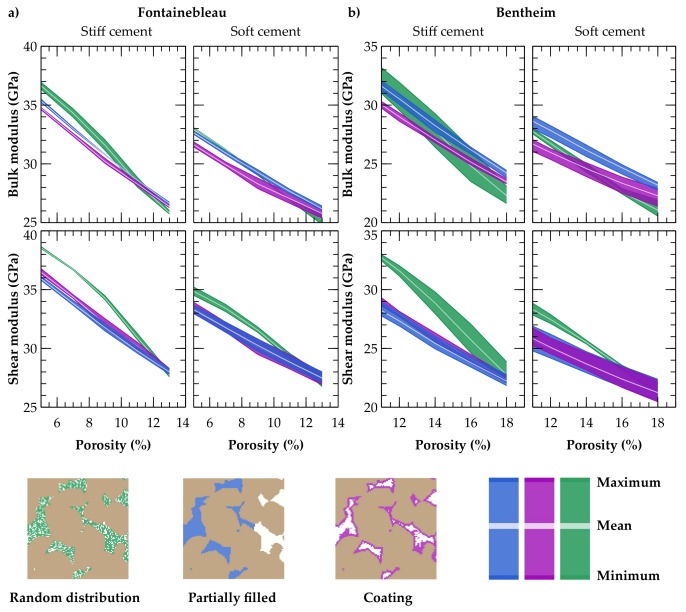
General trends of reduction in elastic properties due to calcite cement dissolution, considering stiff and soft cement moduli for the two investigated digital samples of (**a**) the Fontainebleau and the (**b**) Bentheim sandstones. The range for the three investigated spatial calcite distributions (green, blue, magenta) is based on the three selected representative sub-cubes and determined by their least porosity.

Digital rock weakening by successive dissolution of calcite cement is implemented by removing calcite minerals in our models. For that purpose, porosity-dependent reduction in elastic rock properties is investigated for three spatial cement distributions (Figure 8). The illustrated range is based on the three selected representative sub-cubes and determined by the least porosity of the representative sub-samples. The respective variations are mainly based on the different volume fractions of calcite cement in the sub-volumes due to their different initial porosities. Calculated elastic moduli for a given mineral composition differ only slightly for the three assessed spatial distributions. Due to the simplified partial filling, rocks behave stiffer in parallel direction to the calcite front and weaker perpendicular to it. Nevertheless, partial mineral filling as well as coating result in similar dissolution-induced elastic moduli reductions and differ by 1.9 GPa in maximum for the Bentheim digital sample (Figure 8b). On the contrary, the random calcite distribution shows slightly higher variations in calculated elastic properties with up to 4 GPa and a generally stiffer behaviour, which is reversed for low calcite volume fractions. For both investigated rock samples, the general trends of reduction in bulk and shear moduli resulting from the spatial distribution of the calcite cement are almost identical. However, the Bentheim rock sample exhibits an overall weaker behaviour and broader variation bandwidths due to the higher initial porosities of the three chosen sub-cubes. The decrease in elastic properties of the Bentheim sandstone is slightly lower compared to that of the Fontainebleau sample in view of the absolute moduli reduction at the same relative calcite cementation. Mean differences in bulk and shear moduli of 1.3 GPa are relatively small, but indicate that the impact of cementation is more pronounced at lower porosities, i.e., for the Fontainebleau sample used here.

### 3.3. Impact of Calcite Cement Modulus on Effective Elastic Rock Properties

As expected, applying significantly softer moduli for the calcite cement, whereby the initial stiffness is reduced by 47 %, leads to lower effective elastic rock properties (Table 1). However, the general trend remains mainly unchanged (Figure 8). Only the differences between the three investigated spatial calcite distributions decrease, while the range based on the three representative sub-cubes becomes broader, since differences in their initial porosities become more dominant. Considering a stiff cement, calcite dissolution of 10% by volume out of the pore network reduces bulk and shear moduli in maximum by 34.2% and 38.4%, respectively. Reduction in effective elastic properties amounts to 24.3% (K) and 29.1% (G), assuming significantly softer calcite cement moduli (Table 4). Similar results are obtained for a calcite cement removal of 5% by volume, whereby the differences between soft and stiff cements are less pronounced due to the overall lower calcite volume fraction. Hence, initial elastic parameters of the cement are not the dominant factor, since also considerably softer calcite moduli induce a significant effective elastic property reduction.

## 4. Discussion

For the considered changes in microstructure of both sandstones, porosity is the dominant parameter determining the effective mechanical rock properties, as emphasised by our results indicating only low differences in elastic moduli between the three investigated spatial calcite distributions. Only the random distribution exhibits higher bulk and especially shear moduli, resulting from the interconnection of calcite cement within the pore network, strengthening the entire sample structure. This interconnection decreases for significantly lower calcite volume fractions, whereby more isolated cement minerals occur, resulting in lower effective moduli. The considered random and coating calcite distributions represent two uniform dissolution patterns [81], while synthetic partial filling is an equivalent to a discrete dissolution front. Besides these three scenarios, evolving wormholes or fingering are further observed phenomena [82,83] due to development of preferential flow paths. Even if not taken into account here, it can be assumed that wormholes will induce a significant anisotropy within the microstructure, while the mean value of the effective elastic properties is likely identical to that in the scenarios considered in the present study, e.g., as for the extreme case of partial filling. Examining such complex structures is of particular relevance when anisotropic changes in elastic properties are addressed, which is out of the scope of the present study. Further, our simulation results show slight differences in elastic moduli for all scenarios due to the initial porosity of the sandstones, indicating that the impact of calcite cementation is more pronounced at lower porosities, since cements generally tend to stabilise the rock matrix. This is consistent with the findings of Vanorio et al. [84], who analysed salt precipitation and demonstrated that high-porosity sandstones (porosities > 15%) are less sensitive to rock strengthening than low-porosity ones.

Simulating effective rock properties due to variations in the volumetric calcite cement content by three spatial calcite distributions further allows to quantify rock strengthening due to calcite precipitation. In this context, rock weakening due to mineral dissolution is of particular interest in geological subsurface utilisation, especially related to reservoir, caprock and fault integrity, but also in material sciences related to hydraulically conductive materials (e.g., natural stones used in construction works). Previous studies investigated this effect by means of laboratory experiments [5,6] and numerical simulations [56,75]. While Bemer et al. [5] demonstrated by flow-through experiments on a limestone that homogeneous chemical alterations induced a porosity reduction by up to 2% and a decrease in elastic moduli by up to 35%, Shulakova et al. [56] numerically simulated changes for the same rock type based on micro-CT scans before and after dissolution experiments. They determined a porosity increase of 6.6% by volume and a comparably lower decrease in elastic moduli by 28.6% (K) and 23% (G). The precipitation of 2% salt by volume in sandstones has been experimentally investigated by Vanorio et al. [84] and exhibits an increase in rock stiffness by 4% and 28% for the bulk and shear moduli, respectively. Lamy-Chappuis et al. [6] examined the dissolution of calcite minerals in a sandstone, where an increase in total porosity by only 4.5% reduces rock stiffness by 36% (K) and 33% (G). Following our findings, an absolute porosity decrease by 5% reduces bulk and shear moduli by up to 26.2% and 18.4%, respectively (Table 4). These results are comparable to those of Shulakova et al. [56], but underestimate available experimental data, which is partly related to differences in the rock microstructure, initial porosity and rock property variations. However, the higher elastic moduli reductions determined in the aforementioned laboratory experiments [5,6,84] emphasise the relevance of chemical-mechanical interactions as well as the substantial requirement to improve the related process understanding and to consider these processes in assessments of elastic rock properties.

**Figure 9:**
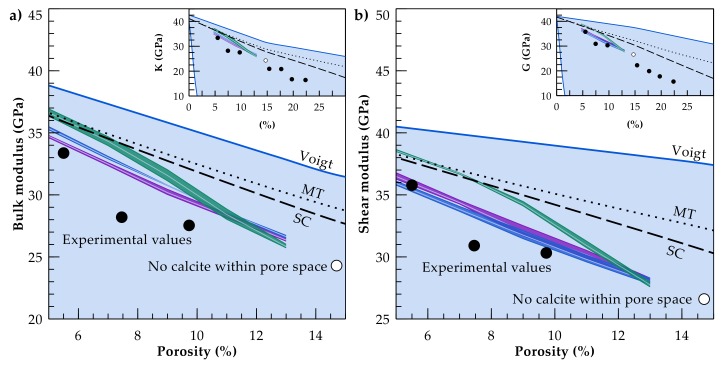
Simulations considering random (green), partially filled (blue) and coated (magenta) calcite distributions substantially reduce the overestimation produced by state-of-the-art analytical solutions as the Mori-Tanaka approach (MT, dotted line) and the self-consistent scheme (SC, dashed line). (**a**) Bulk and (**b**) shear moduli for the Fontainebleau sandstone determined in laboratory experiments [64] (black-filled circles), taking into account only the quartz component and void volume.

Nevertheless, the quantified changes in effective mechanical rock properties can only achieve the quality of the geometrical representation of the initial microstructure. While the elastic moduli determined for the Fontainebleau sandstone exhibit a generally good agreement with available benchmark data [59] and only low overestimation of experimental data (Figure 9), the simulated moduli for the Bentheim microstructure are significantly higher than the published data with 12.5 GPa (K) and 13 GPa (G) [63]. The Bentheim sandstone has minor components of the weak clay mineral kaolinite with 3% by volume [65]. This was not considered in the initial microstructure but could notably reduce elastic rock properties, especially when these are present as pore-lining phase [48]. Moreover, effective elastic moduli overestimation is a general problem within the field of digital rock physics [58] due to the high sensitivity to micro heterogeneities such as cracks, grain contacts and microporosity, which are only insufficiently represented at grain scale [60]. However, improving numerical simulation approaches to determine effective elastic properties is in the scope of ongoing research [58,60,63].

Despite these general challenges in digital rock physics, the method introduced here allows to quantify changes in elastic rock moduli with an increase in accuracy compared to state-of-the-art analytical methods, e.g., the Mori-Tanaka approach or the self-consistent scheme (Figure 9). Furthermore, with the availability of micro-CT scans and/or synthetic microstructures, numerical models allow us to overcome the limitation of using ellipsoidal inclusions, which is generally the case for analytical approaches. Hence, uncertainties related to the composition and structure of the pore network can be quantified in addition to the effect of elastic moduli of cementing minerals as well as temporal and spatial changes in the rock microstructure due to chemical alterations.

## 5. Conclusions

The presented approach is employed to numerically determine bulk and shear moduli of two digital rock samples, the Fontainebleau and the Bentheim sandstones, based on micro-CT images by means of representative sub-cubes. It allows for quantification of changes in mechanical rock behaviour as a result of variations in mineral composition and microstructure, as demonstrated here for the successive removal of calcite cement. Moreover, it enables to determine uncertainties related to porosity variations of micro-CT scans, the effect of initial cement moduli and varying spatial distributions of calcite cement within the pore network. The accuracy in predicting elastic rock properties is substantially improved compared to the general analytical methods. For the dissolution of 10% calcite by volume investigated in the present study, the elastic properties decrease by up to 34% and 38% for the bulk and shear moduli, respectively. For the considered changes in the sandstone microstructure, porosity is clearly the dominant parameter, while significant softer calcite cement moduli lead only to a slightly weaker mechanical behaviour. Also, the spatial cement distribution has only a minor impact, except for the case of a random mineral distribution. Here, the calcite cement is interconnected and stabilises the rock matrix.

Future work will focus on the integration of experimentally-determined variations in mechanical rock behaviour with simulations based on micro-CT images derived from flow-through experiments taking into account chemical reactions. In this context, micromechanical approaches may offer an opportunity to study variations in rock anisotropy, which is of high practical interest. Further, the validation of digital rock physics as potential upscaling methodology for mechanical rock properties is currently intensively discussed in the digital rock physics community. Particularly the choice of the representative volume element size for heterogeneous rock structures, depending on the scope of application is a challenge of high priority. Moreover, different rock types, also including such with pre-existing fractures, should be investigated prioritised by their relevance to geological subsurface utilisation. All these scientific challenges will significantly contribute to the understanding of macro-scale natural phenomena and further improve geotechnical applications, where changes in elastic properties are of particular relevance regarding reservoir, caprock and fault integrity, but also beyond.

## Figures and Tables

**Table 1:** Bulk (K) and shear (G) moduli for selected minerals to compute effective elastic properties.

Mineral	K (GPa)	G (GPa)	Reference
Quartz	37.0	44.0	[27]
Calcite	74.8	30.6	[27]
Calcite (amorphous)	35.5	14.0	[74]
Pore space *	1 × 10 ${}^{-4}$	3 × 10 ${}^{-6}$	

* negligibly small moduli are applied to represent porosity in Code_Aster.

**Table 2:** Bulk (K) and shear (G) moduli of both digital sandstone samples and their correlation to porosity considering all sub-cubes.

Digital Sample	Modulus of Elasticity	Mean Value (GPa)	Mean Directional Difference (GPa)*	r^2^ (Mean Value)	r^2^ (Three Spatial Directions)
Fontainebleau	K	24.8	4.7	0.90	0.68
	G	26.1	4.6	0.94	0.78
Bentheim	K	20.4	9.3	0.84	0.65
	G	19.6	9.5	0.93	0.79

* difference between the maximum and minimum bulk and shear moduli for the three spatial loading directions.

**Table 3:** Porosity as well as bulk (K) and shear (G) moduli of the representative sub-cubes.

Digital Sample	Porosity (%)	Effective Elastic Property		Mean Directional Difference	
		K (GPa)	G (GPa)	K (GPa)	G (GPa)
Fontainebleau	13.9	25.8	27.3	3.0	3.9
	14.6	24.8	26.2	1.6	2.9
	15.6	23.8	25.0	4.6	3.6
Bentheim	19.4	22.4	21.4	2.7	7.0
	21.8	20.7	18.7	5.3	4.0
	24.5	18.2	16.9	2.9	5.0

**Table 4:** Percentage reduction in bulk (K) and shear (G) moduli resulting from the removal of calcite.

Digital Sample	Spatial Cement Distribution	10% Calcite Cement by Volume				5% Calcite Cement by Volume			
		Stiff Cement		Soft Cement		Stiff Cement		Soft Cement	
		K (%)	G (%)	K (%)	G (%)	K (%)	G (%)	K (%)	G (%)
Fontainebleau	Random	32.9	32.8	25.5	25.6	21.9	19.1	18.2	15.6
	Partially filled	30.5	28.0	24.6	22.1	19.0	17.0	15.2	13.4
	Coating	29.3	29.1	22.2	22.5	18.5	17.3	14.6	13.8
Bentheim	Random	34.2	38.4	24.3	29.1	23.2	22.9	18.4	18.1
	Partially filled	33.6	28.7	27.1	22.0	17.4	15.8	14.0	12.6
	Coating	30.2	30.3	21.9	22.4	16.2	16.8	12.2	13.2

## Appendix A. Analytical Methods to Calculate Effective Elastic Properties

Effective elastic rock properties mainly depend on the volume fractions and mechanical properties of their components (minerals and pore fluids) as well as the rock microstructure. The four well-known analytical approaches for calculating effective elastic rock properties, explained in the following were used for comparison against our simulation results.

### Appendix A.1. Voigt and Reuss Bounds

The simplest bounding methods to calculate effective elastic moduli of a composite can be derived from Voigt [21] and Reuss [22]. The authors predict upper and lower strength bounds by averaging elastic parameters of the respective constituents, while neglecting the microstructure. Effective moduli for any given volume fraction fall between these bounds. Voigts average (Equation (A1)) gives the upper bound for the effective elastic properties of a rock, consisting of *i* isotropic components by calculating the arithmetic mean ($K_{Voigt}$) of the component’s moduli ($K_{i}$) weighted by their volume fractions ($f_{i}$).

(A1) $$K_{Voigt}=\sum_{i=1}^{n}K_{i}f_{i}$$

For any composition, Reuss’ average (Equation (A2)) gives the lower bound by determining the harmonic mean ($K_{Reuss}$) of the component’s moduli ($K_{i}$) weighted by their volume fractions ($f_{i}$). The predicted effective elastic properties coincide with the Hashin–Shtrikman lower bound [23], when one of the constituents is a pore fluid with a zero shear modulus [27].

(A2) $$K_{Reuss}={\left(\sum_{i=1}^{n}\frac{f_{i}}{K_{i}}\right)}^{-1}$$

### Appendix A.2. Mori-Tanaka Scheme

The Mori-Tanaka scheme [35,85] belongs to a group of analytical mean-field homogenization approaches, which consider a simplified microstructure in terms of an ellipsoidal shape of its components. It is used for moderate inclusion volume fractions of 30% in maximum [37]. Each of the ellipsoidal inhomogeneities is assumed to be ebedded in an infinite matrix material, derived from the approximations of Eshelby [32]. Interactions between the components are taken into account by applying an effective uniform matrix strain to each of the inclusions. The Mori-Tanaka approach coincides with the Hashin-Shtrikman upper and lower bounds in case of binary mixtures with spherical inclusions [86]. The effective elasticity tensor ($C_{MT}$) of a composite can be calculated by Equation (A3), considering the stiffness of a matrix material ($C_{0}$), its inclusions ($C_{i}$) and the inclusion’s volume fractions ($f_{i}$).

(A3) $$C_{MT}=C_{0}+\sum_{i=1}f_{i}\left(C_{i}-C_{0}\right)A_{i}$$

The strain-localization tensor ($A_{i}$) is given by Equation (A4) and comprises the fourth-order identity tensor (*I*), the volume fractions of the matrix ($f_{0}$) and the influence tensor ($T_{i}$).

(A4) $$A_{i}=T_{i}{\left[f_{0}I+\sum_{j=1}f_{j}T_{i}\right]}^{-1}$$

To determine the influence tensor (Equation (A5)), the Eshelby tensor ($S_{i}$) has to be calculated, which in turn depends on the shape of the ellipsoid and Poisson’s ratio of the matrix ($\nu_{0}$).

(A5) $$T_{i}={\left[I+S_{i}C_{0}^{-1}\left(C_{i}-C_{0}\right)\right]}^{-1}$$

Here, a spherical shape is assumed for all inclusions, whereby the fourth-order Eshelby tensor has the compact form given in Equation (A6). Mathematical expressions for several ellipsoidal inclusion shapes can be found in Mura [87]. The indices ijkl denote the position of the tensor component. Kronecker delta (δ) is equal to one, if two indices have the same value (e.g., $\delta_{ij}=1$ for i=j), otherwise it becomes zero (e.g., $\delta_{ij}=0$ for i≠j).

(A6) $$S_{ijkl}=\frac{5v_{0}-1}{15(1-v_{0})}\delta_{ij}\delta_{kl}+\frac{4-5v_{0}}{15(1-v_{0})}\left(\delta_{ik}\delta_{jl}+\delta_{il}\delta_{jk}\right)$$

### Appendix A.3. Self-Consistent Scheme

Another mean-field homogenization approach is the self-consistent scheme by Hill [34]. It is also based on Eshelby’s solution for an ellipsoidal inclusion embedded in an infinite matrix material and is defined by Equation (A7). Unlike the Mori-Tanaka approach, it can be employed for higher volume fractions [38], since interactions between the components are approximated by replacing the matrix ($C_{0}$) by a material of unknown stiffness ($C^{*}$).

(A7) $$C_{SC}^{*}=C_{0}+\sum_{i=1}f_{i}\left(C_{i}-C_{0}\right)T_{i}$$

Hence, the influence tensor (Equation (A8)) depends on the elasticity tensor of the unknown matrix material ($C_{SC}^{*}$), whereby an iterative implementation is required to calculate $C^{*}$ and the related Eshelby tensor $S_{i}^{*}$.

(A8) $$T_{i}={\left[I+S_{i}^{*}C^{*-1}\left(C_{i}-C^{*}\right)\right]}^{-1}$$
